# Experimental study on the effect of chlorhexidine gluconate (CG)-induced atrial fibrillation on renal water and sodium metabolism

**DOI:** 10.1038/s41598-023-30783-w

**Published:** 2023-03-10

**Authors:** Shuyu Li, Heng Pei, Yaomeng Huang, Da Liu, Liqun Yang, Qi Zhang, Zhijun Wang

**Affiliations:** 1 Department of Cardiology, Affiliated Hospital of North China University of Science and Technology, No. 73 Jianshe South Road, Lubei District, Tangshan, 063000 China; 2 Department of Biochemistry and Molecular Biology, The First Hospital of Hebei Medical University, Shijiazhuang, China; 3 Department of Cardiology, The First Hospital of Hebei Medicical University, Shijiazhuang, Hebei China; 4 Department of Hepatobiliary Surgery, Tangshan Fengnan District Hospital, Tangshan, China; 5 Department of Biochemistry and Molecular Biology, KaiLuan General Hospital, Tangshan, China

**Keywords:** Cardiovascular biology, Experimental models of disease

## Abstract

To construct an animal model of atrial fibrillation and observe the effect of acute atrial fibrillation on renal water and sodium metabolism in mice. A total of 20 C57 mice were randomly assigned to 2 groups (n = 10/group): control group (CON) and atrial fibrillation group (AF). The mice model of atrial fibrillation was induced by chlorhexidine gluconate (CG) in combination with transesophageal atrial spacing. The urine of the two groups of mice was collected, and then we calculate the urine volume and urine sodium content. The expression of TGF-β and type III collagen in the atrial myocardium of the two groups was detected by immunohistochemistry and Western Blot. The levels of CRP and IL-6 in blood were observed by ELISA, and the NF-κB, TGF-β, collagen type III, AQP2, AQP3, AQP4, ENaC-β, ENaC-γ, SGK1 and NKCC proteins in the kidneys of the two groups of mice was observed by Western Blot. Compared with CON, the expression of TGF-β and type III collagen in the atrial myocardium of the mice in AF were increased, the levels of CRP and IL-6 in the blood in AF were increased, and the renal NF-κB, TGF-β, type III collagen AQP2, AQP3, ENaC-β, ENaC-γ, SGK1 and NKCC protein expression in AF were up-regulated. The level of urine volume and urine sodium content in AF were significantly reduced. In the acute attack of atrial fibrillation, the formation of renal inflammatory response and fibrosis is activated, and the renal water and sodium metabolism is hindered, which is related to the up-regulated of the expressions of renal NKCC, ENaC and AQPs.

## Introduction

Atrial fibrillation (AF) is a commonly encountered arrhythmia in clinical practice, and a major predisposing factor for heart failure. Despite significant progress in its treatment, morbidity, disability and mortality associated with AF continue to be high^1,2^. Numerous studies have implicated atrial fibrosis, sympathetic activation, reentry trigger, oxidative stress and inflammation in the occurrence and progression of AF. The reentry trigger leads to the onset of AF, while sympathetic activation provides the basis for electrical remodeling. Atrial interstitial fibrosis disrupts and blocks excitation conduction between cardiomyocytes, and myofibroblasts have been shown to have a direct impact on the development of atrial arrhythmias. This structural remodeling sets the pathological basis for the onset and maintenance of AF, while the inflammatory state promotes the degeneration and fibrosis of atrial myocytes, alters the electrophysiological characteristics of cardiomyocytes, and increases susceptibility to AF. Inflammation is a systemic response, and a significant number of studies have identified inflammatory markers in circulating blood of AF patients^3^. The transforming growth factor β (TGF-β), as an inflammatory factor, is a pivotal growth factor that promotes fibrosis, stimulates fibroblast proliferation and differentiation, increases collagen expression, and encourages fibrosis progression. Nuclear transcription factors are also important in regulating inflammation. For instance, the nuclear factor-kappaB (NF-κB) is activated by inflammatory cytokines, resulting in nuclear translocation, leading to a significant increase in expression of inflammation-related genes and promoting an inflammatory response^4^. Although many studies have reported that AF is associated with a high risk of stroke, heart failure and mortality, few studies have examined the association between acute AF attack and peripheral organ damage, such as kidney damage. Therefore, our study aimed to induce AF in mice model using chlorhexidine gluconate (CG), and to observe whether the acute attack of AF could activate and maintain the formation of renal inflammation and fibrosis, and affect water and sodium metabolism at the physiological and molecular level.

## Experimental methods

Experimental animals and grouping: 20 C57 male mice (18–22 g) were provided by Beijing Huafukang Biotechnology Co., Ltd. They were given clean drinking water and kept in indoor temperature 22–24 °C, humidity 50–60%, light cycle 12 h, light and dark were reared alternately and divided into cages. And they were randomly divided into 10 mice in CON and 10 mice in AF. All animal experiments were conducted in accordance with the guidelines of the Institutional Animal Care Committee, and experiments were conducted in accordance with the Arrival Guide. The animal experiment protocol was approved by the Animal Ethics Committee of Hebei Medical University (Approval Number: 20210366).

## Main reagents

The main reagents are chlorhexidine gluconate stock solution (McLean), pentobarbital (provided by the Chemistry Center Laboratory of Hebei Medical University), CRP and IL-8 ELISA kit (Beijing Solarbio company), immunohistochemical kit ( Beijing Zhongshan Jinqiao), BCA protein quantification kit (Beijing Solarbio Company), rabbit anti-GAPDH (Servicebio Company), rabbit anti-TGF-β (Servicebio Company), rabbit anti-NF-κB(Genetex Company), rabbit anti-collagen III (Servicebio Company), rabbit Anti-NKCC (Abclonal Company), rabbit anti-ENaC-α (Stressmarq Company), rabbit anti-ENaC-γ (Affiniti Company), rabbit anti-SGK1 (Huaan Biotechnology Company), rabbit anti-AQP2 (Abcam Company), rabbit anti-AQP3 (Abclonal Company), rabbit anti-AQP4 (Proteintech).

## Main instruments

PowerLab biological signal acquisition and analysis system (ADINSTRUMENTS company), Scisense catheter electrode (Transonic company), XD-5A cardiac electrophysiological stimulator (Suzhou Industrial Park Dongfang Electronic Instrument Factory), small animal ventilator (Riveworld Life Technology Co., Ltd. company).

## Experimental method

### Establishment of chlorhexidine gluconate model in mice

Anesthesia was induced in the mice by a peritoneal injection of 3% pentobarbital (50 mg/kg). Once an appropriate level of anesthesia was achieved, the mice were placed in a supine position and secured with medical tape on a wristband. The breast area was sanitized using 75% alcohol and the skin was shaved. An oral endotracheal tube was inserted, and the small animal mechanical ventilation was set at a frequency of 100 times/min, with a tidal volume of 2.5 ml and an inspiration-to-expiration ratio of 1:2. The skin, subcutaneous fascia, major pectoral, minor pectoral, and intercostal space were dissected layer by layer to expose the heart. The lungs were protected with small cotton balls, and the pericardium was carefully removed. Sterile CG was gently and evenly applied to the left and right atrium and atrial appendage. After removing the cotton balls, the chest was closed layer by layer. The mice were then placed on a temperature-controlled pad, and the endotracheal tube was removed once the mice had regained consciousness. Following the completion of the experiment, all devices were cleaned and sanitized. In the control group (CON), the mice were subjected to thoracotomy without pericardial tearing and CG application. No deaths occurred in the CON group, while one death occurred in the experimental group (AF).

### Cardiac electrophysiological indexes of mice were detected and recorded through esophageal pacing

On the fourth day following the establishment of the CG-induced atrial fibrillation (AF) model, the mice were anesthetized with 3% pentobarbital (50 mg/kg) and maintained at a body temperature of 37 °C. The Scisense Millar 1.1F electrophysiological catheter electrode was inserted into the esophagus near the left atrium, and a II limb lead body surface electrocardiogram (ECG) was recorded using a computerized data acquisition system. Atrial fibrillation induction was tested with 5-s pulses through the catheter electrode, using high-frequency stimulation S1S2 at a frequency of 50 Hz. After a 5-min stabilization period, the electrical stimulation was repeated three times. Atrial fibrillation induction was considered successful if two out of three stimulation tests resulted in AF. Successful AF induction was defined as a rapid and irregular atrial rhythm lasting at least one second. Transesophageal stimulation was performed in all mice, including those in the control group (CON) and the experimental group (AF). No animal mortality occurred during AF induction.

### Collection of blood, urine and sample of heart and kidney

Urine Collection: Following induction, mice were individually placed in metabolic cages and allowed to feed and drink ad libitum for 12 h. Urine volume was collected over this period.

Collection of Blood, Heart, and Kidney Samples: After 12 h of feeding, the mice were anesthetized with 3% pentobarbital, and the abdominal cavity was opened to collect blood via the inferior vena cava using a coagulation tube. The collected blood was kept at 4 °C. The kidneys and heart were quickly removed, cleaned with ice-cold normal saline, and dried with filter paper. The left atrial appendage was identified, and the left atrial tissue was separated below the left atrial appendage. For each group, the left ear tissue from four animals was partially transformed into paraffin wax samples, and the remaining tissue was stored with liquid nitrogen. The kidney tissues were also stored with liquid nitrogen.

### Enzyme-linked immunosorbent assay (ELISA)

The blood CRP and IL-6 levels were detected by Elisa method, and the experimental procedures were carried out in strict accordance with the instructions of the Elisa kit. The OD value was measured with a microplate reader, and the average value was taken for each group.

### Immunohistochemistry

Immunohistochemical method was used to qualitatively detect the expression of TGF-β and collagen III in the atrium, and the experimental steps were carried out in strict accordance with the instructions of the immunohistochemical kit. The positive expression in the immunohistochemical section is yellow. Under the 200X field of view, 4–5 fields of view are randomly selected from each section for shooting.

### Western blot

Protein lysate (RIPA) reagent was used to extract the total protein of atrium muscle and kidney, and BCA kit was used for protein quantification. Add 20 μg of total protein to the loading buffer, separate with 10% SDS-PAGE gel, transfer to PVDF membrane, block with 5% skimmed milk shaker for 2 h, add primary antibody, incubate overnight at 4 °C with shaker, and then the secondary antibody was incubated at room temperature for 40 min on a shaker. Finally the bands were counted and analyzed using a Bio-Rad gel imager and Image J software.

### Experimental statistical processing

SPSS 23.0 statistical software was used for statistical analysis. The measurement data were expressed as mean ± standard deviation. The measurement data conforming to the normal distribution were analyzed by t test, and P < 0.05 was considered statistically significant.

### Ethics statement

The animal study was reviewed and approved by the Central Laboratory of the first hospital of the Hebei Medical University—the experimental platform.

## Results

### Results 1. The inflammatory response and collagen production of atrial myocytes in CG induced atrial fibrillation model group were increased

**Figure 1:**
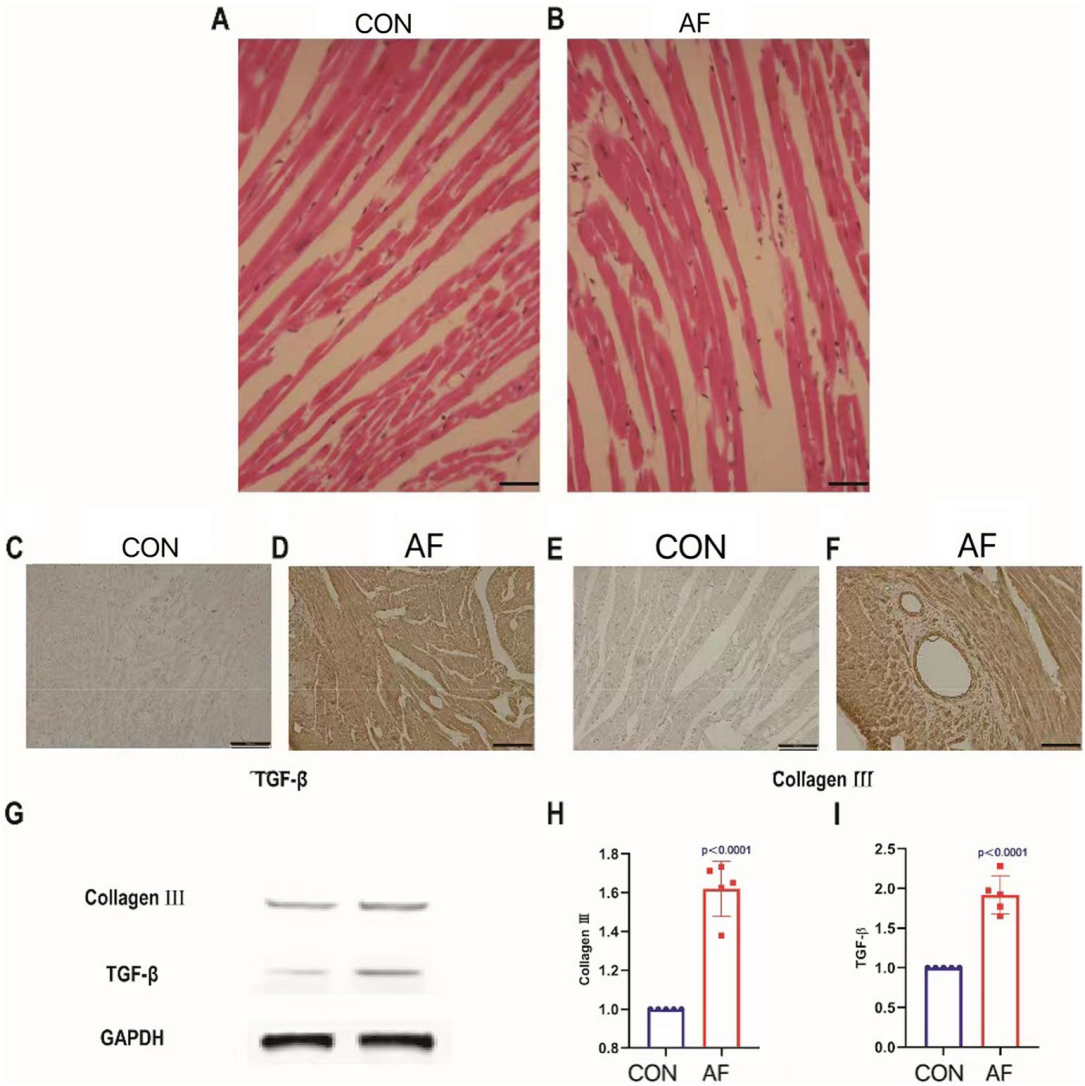
The inflammatory response and collagen production of atrial myocytes were increased in the CG induced atrial fibrillation model group. (**A**) Atrial myocytes histopathologic change in CON was observed by hematoxylin and eosin (HE) staining. (**B**) Atrial myocytes histopathologic change in AF was observed by HE staining. (**C**) TGF-β expression in atrial myocytes in CON was detected by immunohistochemistry. (**D**) TGF-β expression in atrial myocytes in AF was detected by immunohistochemistry. (**E**) Collagen III expression in atrial myocytes in CON was detected by immunohistochemistry. (**F**) Collagen III expression in atrial myocytes in AF was detected by immunohistochemistry. (**G**) Results of Western blotting detecting Collagen III and TGF-β expressions in atrial myocytes in CON and AF. (**H**) Comparison of the expression of Collagen III in atrial myocytes in CON and AF. (**I**) Comparison of the expression of TGF-β in atrial myocytes in CON and AF.

By immunohistochemistry, we observed that the structure of atrial myocytes in the control group was intact, with evenly distributed myocardial fibers arranged both vertically and horizontally (Fig. 1A). In contrast, the atrial myocytes in the group with atrial fibrillation (AF) showed oval or polygonal shapes, with clear cytoplasm, edema, and necrosis, occasional vacuoles, and disordered myocardial fibers. The normal network structure was also lost (Fig. 1B). Moreover, the expression of TGF-β and type III collagen was significantly higher in the AF group than in the control group, as shown by immunohistochemistry (Fig. 1C–F). Quantitative analysis by Western Blot revealed a significant increase in the expression of TGF-β and type III collagen in atrial myocytes of the mice with AF compared to the control group (P < 0.05) (Fig. 1G–I). These results indicate that the CG-induced aseptic atrial myocardium inflammation and fibrosis were successful.

### Electrophysiology

**Figure 2:**
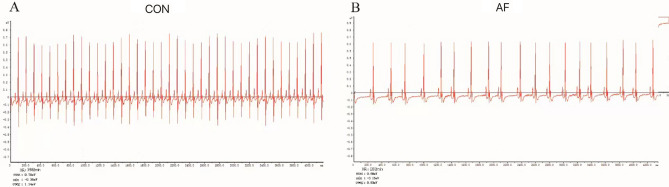
The feature of ECG waveform in two mice groups. (**A**) The feature of ECG waveform in CON mouse. (**B**) The feature of ECG waveform in AF mouse.

The ECG waveform of the mice in CON is shown in (Fig. 2A), and the ECG waveform of the mice in AF is shown in (Fig. 2B). Compared with CON, the ECG of the mice in AF showed no P wave and the arrhythmia was absolutely arrhythmic, which was the ECG manifestation of atrial fibrillation. This indicated that the CG-induced mice atrial fibrillation model was successfully prepared.

### The expression of inflammatory factors in blood, inflammatory response and collagen production in kidney increased in AF mice

**Figure 3:**
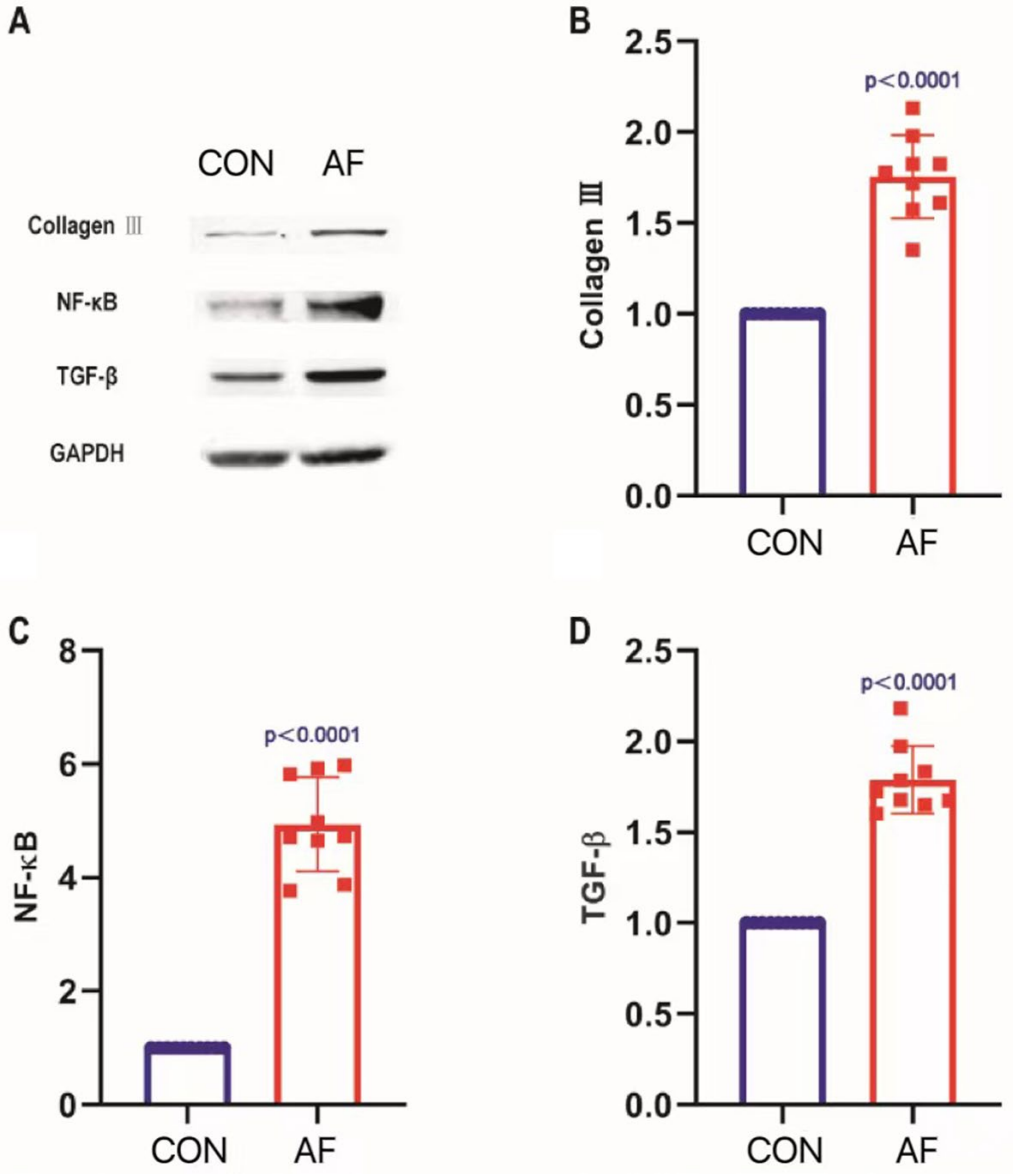
Inflammation and collagen production in mice kidney were increased in AF. (**A**) Results of Western blotting detecting Collagen III, NF-κB and TGF-β expressions in kidneys in CON and AF. (**B**) Comparison of the expression of Collagen III in kidneys in CON and AF. (**C**) Comparison of the expression of NF-κB in kidneys in CON and AF. (**D**) Comparison of the expression of TGF-β in kidneys in CON and AF.

Relative to CON, there was a significant increase in plasma CRP and IL-6 concentrations in AF by ELISA, P < 0.05. (Table 1) This finding indicated that AF acts on systemic inflammatory responses. In comparison with CON, the expression of the NF-κB, TGF-β and collagen type III proteins were significantly higher in the AF group, P < 0.05. (Fig. 3A–D) Which indicated there were a prominent inflammation and fibrosis in kidney in AF.

**Table 1:** Comparison of urine volume, urinary Na + , plasma CRP and IL-6 levels between 2 groups (x ± s).

Group	Urine output (ml/12 h)	Urinary sodium (umol/12 h)	Plasma CRP (μg/L)	Plasma IL-6 (ng/L)
Control group (n = 10)	0.276 ± 0.058	32.840 ± 7.045	154.100 ± 12.76	96.570 ± 4.849
Atrial fibrillation group (n = 9)	0.096 ± 0.043	12.030 ± 5.219	237.600 ± 27.31	131.000 ± 8.017
t-value	7.679	7.244	8.686	11.46
*p*-value	< 0.0001	< 0.0001	< 0.0001	< 0.0001

### Effects of acute onset of atrial fibrillation on renal water and sodium metabolism, renal Aquaporins (AQPs) channels and sodium transporter proteins in mice

**Figure 4:**
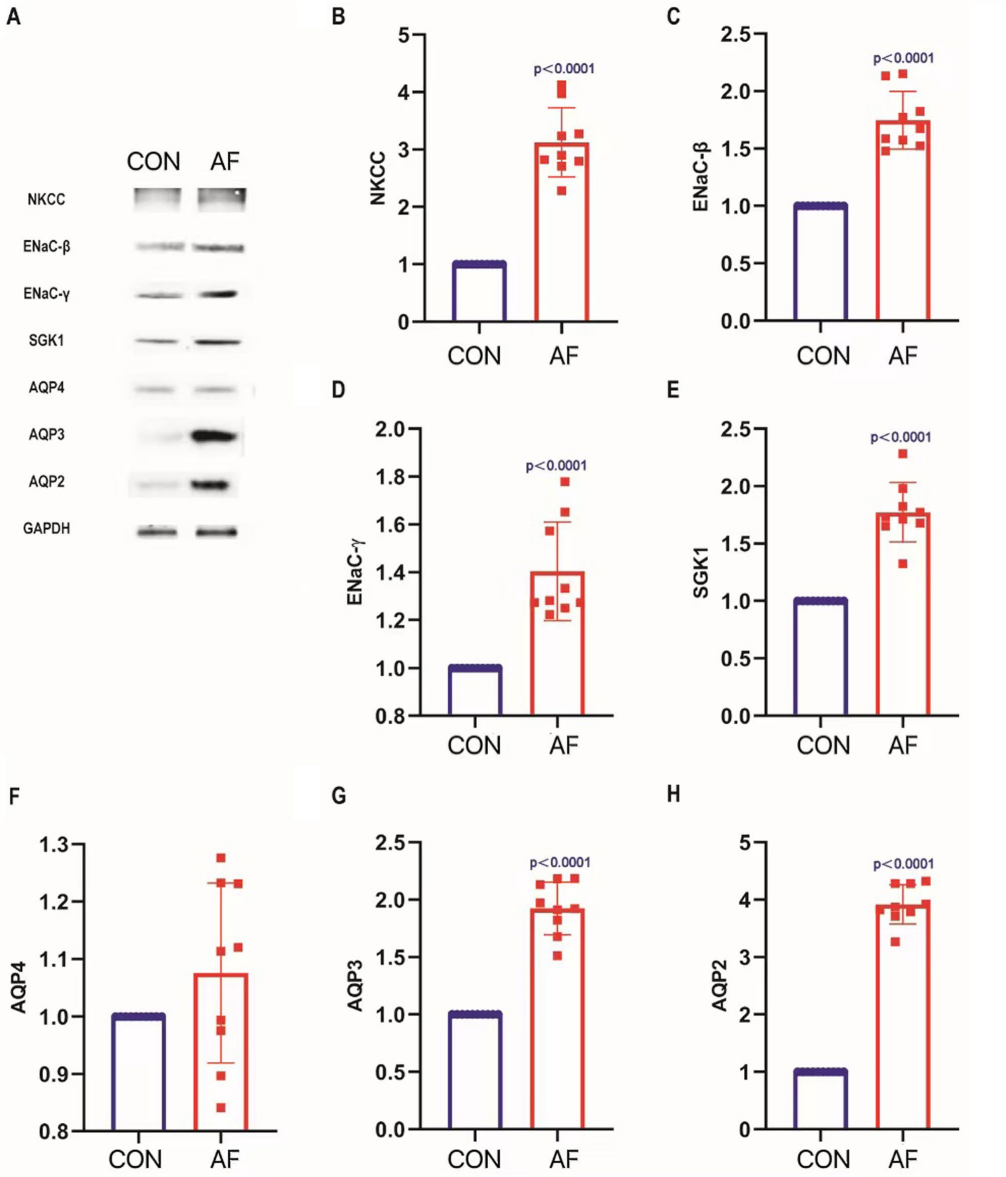
Effects of acute atrial fibrillation on renal water channels and sodium transporters in mice. (**A**) Results of Western blotting detecting NKCC, ENaC-β, ENaC-γ, SGK1, AQP4, AQP3 and AQP2 expressions in kidneys in CON and AF. (**B**) Comparison of the expression of NKCC in kidneys in CON and AF. (**C**) Comparison of the expression of ENaC-β in kidneys in CON and AF. (**D**) Comparison of the expression of ENaC-γ in kidneys in CON and AF. (**E**) Comparison of the expression of SGK1 in kidneys in CON and AF. (**F**) Comparison of the expression of AQP4 in kidneys in CON and AF. (**G**) Comparison of the expression of AQP3 in kidneys in CON and AF. (**H**) Comparison of the expression of AQP2 in kidneys in CON and AF.

Urinary volume and urinary sodium concentrations were measured. We found that, compared with CON, the urine and sodium excretion in AF were significantly reduced, P < 0.05. This was indicating that the water and sodium metabolism of mice in AF was impaired. (Table 1) Relative to CON, the levels of AQP2, AQP3, ENaC-β, ENaC-γ, SGK1 and NKCC proteins expression were increased significantly in AF through Western Blot. P < 0.05. (Fig. 4B–E,G,H). There was no difference in the expression of AQP4 proteins between the two groups. (Fig. 4F). This finding suggested that acute onset of AF affected renal regulation of water and sodium metabolism, which was related to up-regulation of AQPs channels and sodium transporter proteins.

## Discussion

This study found that the onset of atrial fibrillation (AF) was linked to myocardial inflammation and fibrosis. The expression levels of TGF-β and collagen III proteins were higher in AF, along with increased levels of blood inflammatory factors, such as C-reactive protein (CRP) and interleukin-6 (IL-6). Additionally, the expression of renal TGF-β, NF-κB, and collagen III increased, indicating that renal inflammatory response and fibrosis were activated. This, in turn, upregulated the renal sodium transporter proteins and AQPs channels, ultimately impacting renal water and sodium metabolism. Prior research on the effects of acute atrial fibrillation on the kidney from the perspective of inflammation and fibrosis is limited, and this study sheds light on the subject.

Atrial fibrillation (AF), referred to as the “cardiovascular epidemic of the twenty-first century,” arises from various factors, including structural remodeling. This process involves vacuolar degeneration of atrial myocytes, breakage and loss of myogenic fibers, mitochondrial cristae swelling, amyloid degeneration, and atrial interstitial fibrosis caused by collagen aggregation (including degenerated myocardial parenchyma replaced by fibrous tissue). Excessive interstitial fibrosis separates myocardial bundles from each other, and fibroblasts and myofibroblasts couple with cardiomyocytes to interfere with the onset, continuity, and conductivity of electrical signals in cardiomyocytes, thus affecting the self-repair of atrial myocardium. The interaction between disordered atrial fibrillation and structural remodeling plays a crucial role in the development and maintenance of AF^5,6^.

Numerous studies have shown that inflammation stimulates atrial stromal fibrosis^7^. Inflammation not only induces differentiation of atrial fibroblasts into myofibroblasts but also stimulates myofibroblasts to secrete large amounts of collagen. Inflammatory cytokines induce cellular autophagy and apoptosis, which are accompanied by fibroblast recruitment and extracellular matrix deposition. This eventually results in an imbalance in extracellular matrix regulation, manifested by atrial tissue remodeling and stromal fibrosis^8^. This indicates that inflammation and fibrosis can undergo crosstalk and cascade amplification. Therefore, inflammatory animal models have been used to study atrial fibrillation, among which talc-induced atrial fibrillation due to aseptic pericarditis in mice is relatively stable^9^. CG has also been shown to be effective in inducing atrial fibrillation in mice by Yunming Jin et al.^10^. Compared with talc, CG has non-toxic, non-irritating, and stronger antibacterial characteristics, and is often used as a typical inducer of fibrosis models in basic animal experiments. The CG-induced atrial fibrillation model in mice is relatively easy and convenient to operate and has a high success rate; therefore, we chose this model for our study. We found that the expression of TGF-β and type III collagen was significantly increased in the atrial fibrillation group after CG induction. The atrial fibrillation waveform was induced quickly and maintained for a long time, indicating that structural remodeling of the atria had occurred in this atrial fibrillation mice model.

The biological function of TGF-β in promoting inflammation and tissue repair is well-established, with numerous studies confirming its involvement in the structural remodeling of atrial fibrillation^11^. During atrial fibrillation, myofibroblasts and cardiomyocytes release matrix metalloproteinases (MMPs), which degrade the extracellular matrix (ECM) and release cryptic epitopes and TGF-β from the ECM^12^. These cryptic epitopes can interact with cells such as endothelial cells and white blood cells. TGF-β activates the TGF-β/Smads signaling pathway, increasing extracellular matrix accumulation, promoting atrial fibrosis, and ultimately inducing atrial fibrillation^13^.

One characteristic of the inflammatory response is the rise in plasma pro-inflammatory factors. Several studies have established a significant correlation between the occurrence or prognosis of AF and multiple inflammatory markers, such as CRP and IL-6^14–16^. CRP, produced by hepatic cells, is a systemic marker highly susceptible to inflammation. Qiu et al. discovered that salvianolate considerably decreased left atrial enlargement in mice that underwent ligation of the anterior descending branch, lowered serum IL-6 and CRP levels by impeding TGF-β/SMAD-mediated collagen deposition, and thus reduced the susceptibility to atrial fibrillation^14^. This finding confirmed the relationship between CPR, IL-6, and AF in animal experiments. In a subsequent study on 202 AF patients, it was discovered that individuals with higher CRP levels had a higher recurrence rate of AF, implying that CRP was closely linked to the treatment effectiveness of AF^17^. IL-6, a cytokine generated by various cells stimulated by viral inflammation, possesses numerous biological activities in addition to mediating inflammation, such as stimulating fibrinogenic synthesis. Chen’s team found that IL-6 levels were significantly higher in AF patients than in the normal group, and that IL-10 levels were significantly lower. Additionally, IL-6 was discovered to promote the expression of Mir-210 by regulating hypoxia-inducible factor 1α (HIF-1α) in cultured mouse cardiomyocytes. Mir-210 inhibited T cells by targeting Foxp3, promoting atrial myocyte fibrosis^18^. In this study, it was discovered that the expression of atrial type iii collagen and TGF-β increased in the atrial fibrillation group in the CG-induced mouse atrial fibrillation model, while the levels of plasma CRP and IL-6 increased, consistent with the aforementioned findings, indicating that atrial fibrillation, inflammation, and myocardial fibrosis are closely connected. At the same time, the study confirmed that the inflammatory response initiated in the acute episode of atrial fibrillation in mice is systemic.

Increased pro-inflammatory mediators and inflammatory factors can affect renal function. In this study, the expression of NF-κB and TGF-β proteins in the kidneys of mice with atrial fibrillation also increased with an increase in plasma inflammatory factors. The study found that the NF-kappa B as a nuclear transcription factor can be mediated the classic inflammatory signaling pathways, lead to TGF beta activation, the activation and the activation of kidney, glomerular capillary endothelial cells, basement membrane of kidney damage caused directly or indirectly, to increase the accumulation of extracellular matrix and protein loss, promote kidney fibroblast proliferation and fibrosis. Impaired kidney function^19–23^. In this study, the expression of type I collagen was significantly up-regulated in the atrial fibrillation group. As the main component of the extracellular matrix, over-expression of collagen type indicates the appearance of fibrosis^24^. At the same time, it was found that the renal drainage and sodium discharge ability of mice in the AF group decreased, indicating that the renal function of mice in the AF group was affected. Elevated concentrations of AQP2, ENaC-β, ENaC-γ and NKCC were also found. ENaC, namely amiloride-sensitive epithelial sodium channel, is composed of α, β and γ subunits and is expressed in the intracellular vesicles and apical membranes of the main cells of the connective and collecting ducts of the kidney, which is a rate-limiting step of sodium reabsorption and plays a crucial role in controlling systemic sodium balance^25^. Functional ENaC relies primarily on hydrolysis of the γ subunits and phosphorylation of the β subunits for transfer of the endoplasmic reticle to the cell membrane^26,27^. Serum glucocorticoid kinase 1 (SGK1), as a widespread in vivo regulator, plays a major role in the inflammation and metabolism of sodium hydrates. In this study, we found that SGK1, as a direct upstream regulator of ENaC, reflects the increased inflammatory response and sodium hydrate metabolism disorder in mice with acute onset of atrial fibrillation. NKCC, a furosemide-sensitive sodium–potassium-chloride cotransporter, is expressed in epithelial cells of the coarse segment of the romance of the medullary loop and in dense spots, and is mainly involved in saline reabsorption and tubular feedback. NKCC is mainly regulated by AVP system and Natriuretic peptide system^28^. AQP2 and AQP3 are located in apical membrane and the basement membrane of renal tubules, respectively, and their expression level is increased, indicating that renal tubules have increased water absorption^29^. Therefore, it can be inferred that the inflammatory response and fibrosis activation of the kidney up-regulate the expressions of NKCC, ENaC and water channels in the renal epithelium, thus affecting the hydration and sodium metabolism of the kidney.

Several studies have reported that stimulating the production and secretion of atrial Natriuretic peptide (ANP) after the onset of atrial fibrillation can play a diuretic role due to atrial dilatation and traction, as well as sympathetic nerve excitation. However, other studies have suggested that the increase in ANP may not result in a corresponding diuretic effect when the expression of Natriuretic peptide receptor is decreased or desensitized, which can diminish the diuretic effect of ANP^30^. Additionally, Cao et al. observed that as atrial fibrillation progressed, the ANC level initially increased and then decreased^31^. Further research is needed to confirm whether changes in the Natriuretic peptide system occur during the acute atrial fibrillation episode.

Of course, heart failure, the most frequent complication of atrial fibrillation, is also a critical factor that affects kidney function^32^. Atrial fibrosis, as a pathological structural basis of atrial fibrillation, represents a potential association between atrial fibrillation and heart failure^33^. Atrial fibrosis can result from decreased left ventricular myocardial compliance, left ventricular diastolic dysfunction, increased left ventricular filling pressure, and chronic inflammatory response to heart failure^34,35^. Left ventricular diastolic dysfunction can also occur as a result of rapid ventricular response due to atrial fibrillation. Pathological changes such as hemodynamics, neurohormones, and inflammation after the onset of heart failure can cause renal failure in approximately 40–50% of patients with heart failure^36^. In this study, the disorder of renal water and sodium metabolism caused by acute atrial fibrillation may be the initiating factor of heart failure, which predates its occurrence. The acute onset of atrial fibrillation triggers a systemic inflammatory response, leading to an increase in systemic blood volume, while promoting kidney inflammation and fibrosis, resulting in water and sodium metabolism disorders. This mechanism can lead to a significant increase in water and sodium retention, which increases the heart's preload and accelerates the development of heart failure. Additionally, the acute onset of atrial fibrillation may lead to the activation of neurohormones, the activation of the renal renin-angiotensin system, and result in disorders in water and sodium metabolism.

The specific mechanism requires further verification through experiments. Juan et al.^32^ reported a fivefold increase in the risk of renal deterioration in patients with atrial fibrillation after developing heart failure. Studies have demonstrated that glucocorticoids can produce a potent diuretic effect and improve cardiac function by inhibiting the renin–angiotensin–aldosterone system and arginine vasopressin pathway, as well as activating the natriuretic peptide system in mice with heart failure^37^. It remains to be determined whether glucocorticoids can improve inflammation and fibrosis in mice with atrial fibrillation by the aforementioned mechanisms, thereby improving renal function.

This study examined the mechanism of renal damage caused by atrial fibrillation for the first time, but it has several limitations. Firstly, the study only used male C57 mice, which may not reflect sex-specific differences in toxicity or metabolism. Secondly, the study was solely conducted in vivo and did not include in vitro experiments or investigate signal transduction pathways. Additionally, the study only examined the effects of acute atrial fibrillation on renal water and sodium metabolism, and did not explore the effects of persistent atrial fibrillation on the kidneys. The experimental conclusion requires confirmation from clinical research. Finally, the changes in the natriuretic peptide system, which play a diuretic role in atrial fibrillation, require further study for confirmation.Finally, Sp1 has been reported upregulated in glomerulonephritis and is an important transcriptional mediator of TGF-β signaling. And Sp1 was a hub gene of atrial fibrillation^38^. And G protein Signaling (RGS)-4 (RGS4) was found that may protect not only against calcium signaling-induced tachyarrhythmias and AF, but also against cholinergic-induced bradycardia. It was also reported to be potentially elevated in AF in the atrial myocytes of their AF mice recently^39^. In addition, osteopontin (OPN) is a ubiquitous pro-inflammatory cytokine, which is also known to mediate cardiac TGF-β pro-fibrotic and pro-inflammatory signaling that underlies AF^40^. Meanwhile, SGLT2, known to be involved in renal fibrosis and various nephropathies, has been reported to be induced by TGF-β in renal cells^41^. The expression of Sp1, RGS4 and OPN in atrium as well as the expression of OPN and SGLT2 expression in kidney, especially OPN in the medulla and cortex of kidney in mice in acute atrial fibrillation in mice is worth to study further. Future studies are warranted to test.

In conclusion, our study found that the acute onset of atrial fibrillation (AF) triggers an atrial inflammatory response, which in turn activates renal inflammatory response and fibrosis. This, in turn, leads to the dysfunction of renal water and sodium metabolism, which is related to the up-regulation of the expressions of renal NKCC, epithelial sodium channel, and AQPs channel. The findings of this study provide a novel direction for the prevention and treatment of heart failure on a global scale.

## Supplementary Information

Supplementary Information.

## Data Availability

The original contributions presented in the study are included in the article/Supplementary Material, further inquiries can be directed to the corresponding author/s.
