# Supplementary material for: Experimental study on the effect of chlorhexidine gluconate (CG)-induced atrial fibrillation on renal water and sodium metabolism
Source: Sci Rep. 2023 Mar 10;13:4016. doi: 10.1038/s41598-023-30783-w (PMC10006165; doi:10.1038/s41598-023-30783-w)
Supplement: Supplementary file 1 — Supplementary Information. [file 41598_2023_30783_MOESM1_ESM.docx]

Raw Data

Atria Myocardium

This is the original blot of TGF-β in fig3.

1 TGF-β


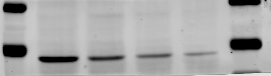


This is the original blot of TGF-β in fig1.

The order of adding samples is AF(atria myocardium ) CON(atria myocardium) AF(kidney) CON(kidney), the reason of the trend is contrary to the figure in the article is the scanning problem, the band was inverted during the scanning. We are very sorry for about this.


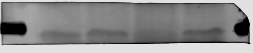


The order of adding samples is CON(atria myocardium) AF(atria myocardium ) CON(kidney) AF(kidney).

2 Collagen III


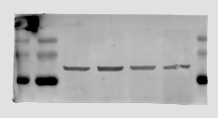


This is the original blot of collagen III in fig1.

The order of adding samples(atria myocardium) is CON1 AF1 CON2 AF2. The bands of second group were scratched.


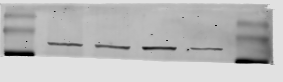


This is the original blot of collagen III in fig3.

The order of adding samples(Kidney) is AF1 CON1 AF2 CON2, the reason of the trend is contrary to the figure in the article is the scanning problem, the band was inverted during the scanning. We are very sorry for about this.


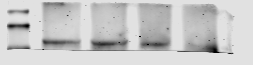


The order of adding samples is CON3(atria myocardium) AF3(atria myocardium) CON3(Kidney) AF3(Kidney), , and the last band was scratched. We are so sorry about this.

3 NF-κB


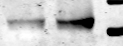


This is the original blot of NF-κB in fig3.

The order of adding samples(Kidney) is CON1 AF1.


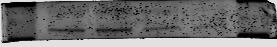


The order of adding samples is CON2(kidney) AF2(kidney) CON1(cortex) AF1(cortex).

4 NKCC


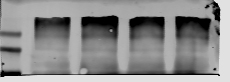


This is the original blot of NKCC in fig3.

The order of adding samples(Kidney) is CON1 AF1 CON2 AF2.


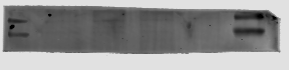


The order of adding samples(Kidney) is CON3 AF3 CON4 AF4, and the bands of the fourth group were scratched.

5 ENaC-β


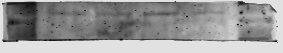


This is the original blot of Enac-β in fig3.

The order of adding samples(Kidney) is AF1 CON1 AF2 CON2, the reason of the trend is contrary to the figure in the article is the scanning problem, the band was inverted during the scanning. We are very sorry for about this.


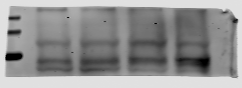


The order of adding samples(Kidney) is CON1 AF1 CON2 AF2.

6 ENaC-γ


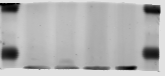


This is the original blot of ENaC-γ in fig3.

The order of adding samples(Kidney) is CON1 AF1 CON2 AF2, and the bands of the first group were scratched.


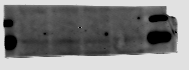


The order of adding samples(Kidney) is CON3 AF3 CON4 AF4, and the last band was scratched. We are so sorry about this.

7 SGK1


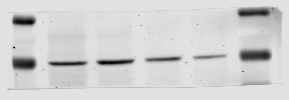


This is the original blot of SGK-1 in fig3.

The order of adding samples is CON1(kidney) AF1(Kidney) CON1(cortex) AF1(cortex).


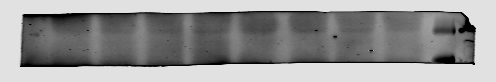


The order of adding samples is CON2(kidney) AF2(Kidney) CON3(kidney) AF3(Kidney) CON2(cortex) AF2(cortex) CON3(cortex) AF3(cortex).

8 AQP4


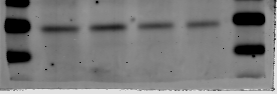


This is the original blot of AQP4 in fig3.

The order of adding samples(Kidney) is CON1 AF1 CON2 AF2.

9 AQP3


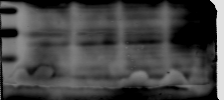


This is the original blot of AQP3 in fig3.

The order of adding samples(Kidney) is CON1 AF1 CON2 AF2.


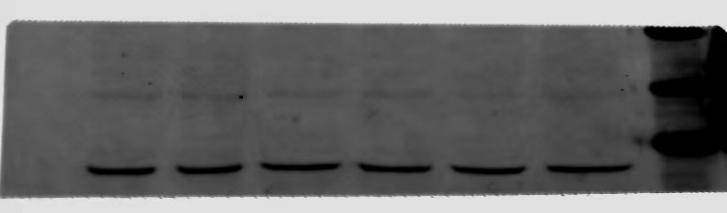


The order of adding samples(Kidney) is CON3 AF3 CON4 AF4 CON5 AF5.

10 AQP2


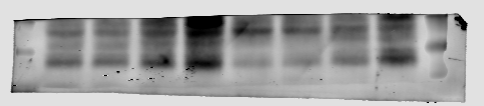


This is the original blot of AQP2 in fig3.

The order of adding samples(Kidney) is CON1 AF1 CON2 AF2 CON3 AF3 CON4 AF4.
